# REVIEW: The evolving linkage between conservation science and practice at The Nature Conservancy

**DOI:** 10.1111/1365-2664.12259

**Published:** 2014-05-19

**Authors:** Peter Kareiva, Craig Groves, Michelle Marvier

**Affiliations:** ^1^ The Nature Conservancy 4722 Latona Avenue NE Seattle WA 91805 USA; ^2^ The Nature Conservancy 40 E. Main Street, Suite 200 Bozeman MT 59715 USA; ^3^ Department of Environmental Studies and Sciences Santa Clara University 500 El Camino Real Santa Clara CA 95053 USA

**Keywords:** biodiversity, conservation, corporate practices, development by design, ecosystem services, education, sustainability

## Abstract

1. The Nature Conservancy (TNC) was founded by ecologists as a United States land trust to purchase parcels of habitat for the purpose of scientific study. It has evolved into a global organization working in 35 countries ‘to conserve the lands and waters on which all life depends’. TNC is now the world's largest conservation non‐governmental organization (NGO), an early adopter of advances in ecological theory and a producer of new science as a result of practising conservation.
2. The Nature Conservancy's initial scientific innovation was the use of distributional data for rare species and ecological communities to systematically target lands for conservation. This innovation later evolved into a more rigorous approach known as ‘Conservation by Design’ that contained elements of systematic conservation planning, strategic planning and monitoring and evaluation.
3. The next scientific transition at TNC was a move to landscape‐scale projects, motivated by ideas from landscape ecology. Because the scale at which land could be set aside in areas untouched by humans fell far short of the spatial scale demanded by conservation, TNC became involved with best management practices for forestry, grazing, agriculture, hydropower and other land uses.
4. A third scientific innovation at TNC came with the pursuit of multiobjective planning that accounts for economic and resource needs in the same plans that seek to protect biodiversity.
5. The Millennium Ecosystem Assessment prompted TNC to become increasingly concerned with ecosystem services and the material risk to people posed by ecosystem deterioration.
6. Finally, because conservation depends heavily upon negotiation, TNC has recently recruited social scientists, economists and communication experts. One aspect still missing, however, is a solid scientific understanding of thresholds that should be averted.
7. *Synthesis and applications*. Over its 60‐plus year history, scientific advances have informed The Nature Conservancy (TNC)'s actions and strategies, and in turn the evolving practice of conservation has altered the type of science sought by TNC in order to maximize its conservation effectiveness.

## Abstract 2

Over its 60‐plus year history, scientific advances have informed The Nature Conservancy (TNC)'s actions and strategies, and in turn the evolving practice of conservation has altered the type of science sought by TNC in order to maximize its conservation effectiveness.

## Introduction

The Nature Conservancy (TNC) was founded by plant ecologists in 1951 for the ‘preservation of natural lands for scientific use’. Over time, the organization has undergone a remarkable transition from protecting lands in the service of science to using science in the service of nature protection. Today, TNC's mission is ‘to conserve the lands and waters on which all life depends’. We focus here on TNC due to its outsized influence on conservation practice. This one organization, with more than one million dues‐paying members and 3800 staff, including some 600 scientists, controls more than one‐fourth of all assets held by a sample of 1743 conservation organizations registered for tax purposes with the U.S. government (Armsworth *et al*. 2012). This sample of conservation non‐profits includes the other major international conservation organizations such as Conservation International (CI), World Wildlife Fund (WWF)‐U.S. and Wildlife Conservation Society (WCS), and a total of 184 organizations with an international focus. Based on FY2011 U.S. tax returns, TNC's revenues were more than 1·5 times those of the next three largest conservation NGOs (CI, WWF and WCS) combined, and TNC's assets were more than four times the combined holdings of these next three largest organizations. TNC has scientists on the ground in all fifty U.S. states and in over 35 countries spanning Africa, Asia, Australia, Pacific Island Nations, South and North America.

Here, we highlight how insights from ecological science, and more recently from the social sciences, have transformed the way TNC does its work. Today, TNC scientists are both consumers and producers of basic ecological knowledge and applied conservation know‐how, collectively publishing more than 200 peer‐reviewed papers each year. Most large environmental and conservation non‐governmental organizations (NGOs) employ respected scientists and use science to inform their actions. What may distinguish TNC, however, is the extent to which being ‘science based’ is seen by its supporters as the top reason they give money to TNC, that its Executive Team includes a scientist reporting directly to the CEO, and the presence of a strong scientific voice at the board level, currently including four members of the U.S. National Academy of Sciences and a Pew Fellow.

## TNC's early focus on ‘Bucks and Acres’

The Nature Conservancy (TNC) had its origins in the Ecological Society of America (ESA), which in the 1940s established the Committee on Preservation of Natural Conditions. This committee sought to preserve natural areas for scientific study. The committee and its director Victor Shelford were at odds with much of the rest of the ESA, which looked unfavourably on the non‐academic objective of protecting land. As a result, the Committee on Preservation decided in 1946 to form their own group independent of the ESA, called the Ecologists' Union (Dexter 1978; Smith & Mark 2009). This group incorporated as a non‐profit organization in 1951 as The Nature Conservancy, with the mission of preserving examples of important ecosystems that could be used for scientific study. At the time of its founding, the notion of using private sector funding to preserve natural areas was a radical idea, one that spawned a remarkable growth in the land trust industry in the United States and eventually globally.

**Figure 1:**
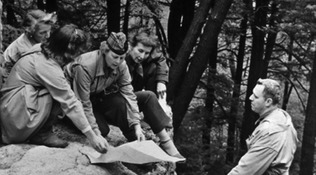
Land acquisition, a key protection tool for TNC, began with a 24‐ha purchase in 1955 along the Mianus River Gorge on the New York/Connecticut border. © The Nature Conservancy.

The Nature Conservancy was an early pioneer in the arena of land acquisition and protection, acquiring its first property – a 24‐ha parcel along the Mianus River Gorge on the New York/Connecticut border – in 1955 (Fig. 1). The organization funded the purchase, but stipulated that the money had to be repaid for use in other conservation efforts. This revolving loan fund was a key innovation that remains in use at TNC today.

Throughout the 1960s, TNC leadership shifted from volunteer scientists to professional finance and business people, and the organization became increasingly business‐like. In 1970, Robert Jenkins was hired as TNC's first ever staff scientist. He reports that when he began working at TNC, ‘the Conservancy was quite small and everyone in it was totally wrapped up with land acquisition, fund‐raising and deal‐making’. Jenkins sought to steer TNC towards a systematic method of prioritizing lands for purchase. Within a few years, he had persuaded TNC ‘to adopt the “preservation of natural diversity” as its mission, the first institution on Earth to do so’ (Jenkins 2010).

## Natural Heritage inventory

Jenkins recognized that to align TNC's purchases with its newly adopted biodiversity mission would require vast amounts of data on the occurrences of species and ecological communities. Under Jenkins' leadership, TNC initiated a Natural Heritage Programme in collaboration with the South Carolina state wildlife agency in 1974 to inventory the state's plant and animal species and ecological communities (Groves, Klein & Breden 1995). Eventually, similar state–TNC partnership programmes spread to every U.S. state and in the early 1980s to nations in Latin America. The innovation of these programmes was to systematically assemble occurrence information by species and community type and then use those occurrence data to identify the areas most in need of protection. An important early idea in systematic conservation planning known as the coarse‐filter–fine‐filter approach emerged from the early work of the Natural Heritage Programmes. Under this approach, occurrences of ecological communities are used as a coarse filter, with the goal of conserving all major plant community types within a region. The occurrences of rare species were then used as a fine filter to hone the portfolio of potential conservation sites (Hunter 1991).

The Natural Heritage Programme grew rapidly in the number of staff, and inventory and mapping of species and community occurrences dominated TNC science for nearly 20 years. The programme generated extensive data on the status and distribution of rare species and ecological communities at the state and national level in the United States, as well as in Canada and much of Latin America. This data base remains widely used by TNC as well as by government natural resource agencies. An outgrowth of this effort was a book entitled *Precious Heritage,* which documented the status of U.S. biodiversity (Stein, Kutner & Adams 2000). In the 1990s, TNC's Board of Directors decided to spin off the Natural Heritage Programmes as a separate organization, which came to be known as NatureServe and became fully independent of TNC in 2000. Among the reasons for this separation was the idea that TNC science should focus on modelling, hypothesis testing and planning tools and leave inventory and data curation to a separate institution.

## Conservation by Design: a more systematic approach to conservation

By the early 1990s, TNC scientists realized that a site‐by‐site approach to conservation that was focused primarily on rare ecological communities and species had a serious limitation – it lacked a broader vision of what efforts were needed to conserve the biodiversity and the underlying ecological processes of entire ecosystems. In response, TNC developed a more comprehensive conservation framework known as *Conservation by Design* (Nature Conservancy 2006). That framework, first published in 1996, led to the development of ecoregional plans and assessments – broad visions of the most important places for conserving the biodiversity of large ecological regions (Groves 2003) and a site‐based strategic planning framework (Poiani *et al*. 1998).

**Figure 2:**
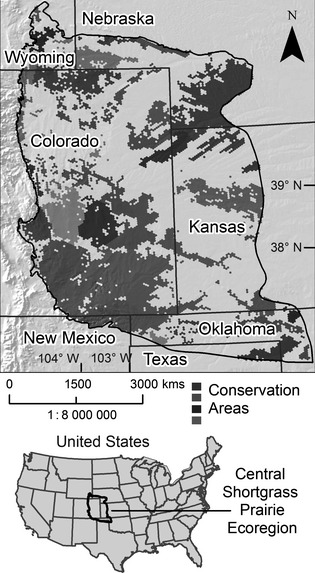
A portfolio of conservation areas resulting from an ecoregional assessment in the Central Shortgrass Prairie Ecoregion of the United States (Neely *et al*. 2006). An early generation version of Marxan, a decision support system known as SPOT or Spatial Portfolio Optimization Tool, was used in conjunction with GIS, to select the conservation areas in this portfolio based on the occurrence of various conservation targets (elements of biodiversity) in the ecoregion and the quantitative goals set for these targets.

Conservation by Design follows an adaptive management framework of (i) setting priorities, (ii) developing strategies, (iii) taking action and (iv) measuring results. Setting priorities has largely been accomplished through ecoregional plans and more recently through Global Habitat Assessments (Hoekstra *et al*. 2005). Ecoregional plans result from systematic conservation planning (Margules & Pressey 2000), and their most important product is a map of putative conservation areas referred to as a ‘portfolio of conservation sites’ (Fig. 2). An important by‐product of ecoregional assessments is the widely used decision support system in conservation planning called Marxan (Ball, Possingham & Watts 2009), which was in part developed to meet the needs of TNC planning teams. While ecoregional assessments might seem an unremarkable innovation, these plans transformed TNC's investments from a largely opportunistic to a highly strategic enterprise. A recent analysis of TNC's land purchases revealed that 86% of acquired properties fall within priority areas identified by science‐based ecoregional plans (Fisher & Dills 2012).

Once priority areas are identified, strategies for protecting those areas must be developed. To meet this need, TNC scientists formalized an approach to site‐based strategic planning referred to as Conservation Action Planning or CAP, which is now widely used inside and outside TNC. The steps of CAP closely parallel those of the Conservation Measures Partnership's Open Standards for the Practice of Conservation (Schwartz *et al*. 2012; Conservation Measures Partnership 2013). The most common outputs of Conservation Action Planning are a threat assessment, conceptual models and results chains that portray a theory of change for a given set of strategies (Margoluis *et al*. 2013).

‘Taking action’ at TNC often implied the traditional workings of a land trust: land protection (e.g. purchasing land outright or placing a conservation easement on land) and ecological stewardship. As TNC acquired more lands in the 1970s, the organization quickly appreciated the need to ecologically manage its preserves and therefore hired land stewards who began experimenting with fire and other restoration management tools. In the late 1980s, the variety of stewardship activities expanded significantly to include grazing management, weed control and even altering flow regimes at dams. At the same time, the spatial scale of TNC's work grew in part because the new field of landscape ecology suggested that larger conservation areas tended to have longer‐term viability and greater ecological integrity (Poiani *et al*. 2000). Freshwater ecologists, hydrologists, fire ecologists, invasive species biologists, landscape ecologists, marine biologists and biological monitoring experts were hired into a central science programme to bring much‐needed expertise and technical support to field programmes. Today, TNC has globally recognized strengths in freshwater ecology, hydrology and marine biology (e.g. Postel & Richter 2003; Opperman *et al*. 2009; Beck *et al*. 2011) as a result of these early investments.

The fourth component – measuring results and evaluating the effectiveness of conservation actions – has been the ‘Achilles heel’ of the conservation community (Legg & Nagy 2006). To partially address this weakness, TNC was a founding member in 2002 of the Conservation Measures Partnership (CMP), an alliance of conservation organizations aimed at improving the practice of monitoring and evaluation. TNC also helped establish and fund one of the most important outgrowths of CMP – the Conservation Coaches Network (http://www.conservationgateway.org/ConservationPlanning/ActionPlanning/Network/Pages/conservation-coaches-netw.aspx ), a group of trained practitioners who help advance best practices of the CMP's Open Standards. In the early 2000s, TNC sought to strengthen ‘monitoring and evaluation’ by encouraging and developing senior management reviews, peer‐review workshops, online training modules, guidance papers and published case studies on monitoring and adaptive management (e.g. Lemke *et al*. 2011). A key lesson has been that monitoring data often are not given much attention by managers, unless they address questions that the managers, as opposed to scientists, want answered (Montambault & Groves 2012). Moreover, managers are reluctant to invest in monitoring because it is sometimes seen as a wasted expenditure. To overcome this reaction, TNC scientists have developed guidance that focuses monitoring investments in projects with the greatest opportunity for learning and leverage, as well as projects that pose significant organizational risks (Montambault & Groves 2012).

## Global habitat analyses and programmatic expansion

The Nature Conservancy, like most environmental NGOs, establishes organization‐wide goals to both inspire and manage towards. In 2004, then President of TNC, Steve McCormick, asked TNC's science staff to complete a global analysis of threats and habitat protection and to produce science‐based goals for conservation to guide TNC for the next decade. Those analyses led to a major book, *The Atlas of Global Conservation* (Hoekstra *et al*. 2010), and dozens of scientific papers. The science‐driven organizational goal that emerged was to protect 10% of all major habitat types by 2015. This was time bound, specific and, by being stratified by major habitat type, escaped the downside of simply focusing on biodiversity hotspots (Kareiva & Marvier 2003).

**Figure 3:**
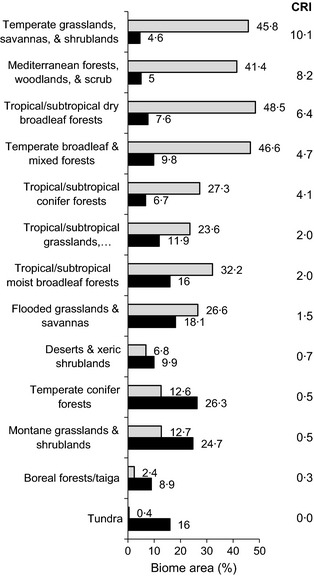
Global habitat loss and protection for 13 major habitat types. The conservation risk index (CRI) is the ratio of habitat converted (grey bars) to protected (black bars). Data are from Hoekstra *et al*. (2005).

These analyses influenced where TNC expanded internationally. In particular, Hoekstra et al. (2005) found that, of all of the world's major habitat types, temperate grasslands had the highest cumulative risk index as measured by the ratio of % habitat converted to % habitat protected (Fig. 3). In other words, little grassland habitat remains compared to its original global extent, and most of what is left is unprotected. As a direct and immediate consequence of this analysis, TNC opened new country programmes in Argentina and Mongolia, countries that harbour the largest remaining examples of intact grassland ecosystems.

## Protected areas are not sufficient

Over the course of its history, TNC has either directly protected through easements and purchase or assisted partners and governments in the protection of 48 million hectares (http://www.nature.org/about-us/index.htm?intc=nature.tnav.about), transferring much of this land to governments or local land trusts. TNC was an early pioneer of the debt‐for‐nature approach to protected area financing, and its first such swap resulted in the 1988 expansion of Costa Rica's Braulio Carrillo National Park. More recently, TNC protected much of the Palmyra atoll, recognized as among the world's most pristine coral ecosystems. And, in collaboration with Trust for Public Land, TNC recently completed the largest ever private land conservation transaction, spending $US 490 million to conserve more than 125 000 hectares of forested land in Montana.

While TNC continues to help establish protected areas, scientists have increasingly recognized that these areas are not fail‐safe. In many cases, poaching and deforestation continue within parks. In response, TNC, with funding from the U.S. Agency for International Development, launched in 1989 the Parks in Peril programme to help improve the management of existing protected areas in Central and South America and the Caribbean. Despite this and related efforts, deforestation within protected areas continues (e.g. Kinnaird *et al*. 2003). For example, a recent TNC‐led study assessed the extent of land and forest degradation between 2004 and 2009 within 1788 Latin American protected areas. Using remote sensing data, Leisher *et al*. (2013) documented that 45% of the examined protected areas had experienced degradation and deforestation on more than 1 million hectares. Moreover, a growing number of efforts around the world seek to downgrade, downsize and even degazette protected areas (Mascia *et al*. 2014). Hunger for minerals and fossil fuels is a major driver of these efforts but so is pressure from rural people cut‐off from the natural resources they had previously relied upon.

Purchasing lands or easements for protection and creating parks are important and enduring strategies. At the same time, TNC recognized that protected areas can only achieve a fraction of what is needed for biodiversity conservation and that complementary strategies are also needed (Kareiva 2014). TNC has therefore broadened its focus to include multiple‐use landscapes and seascapes, in addition to more strictly managed conservation areas. This shift spurred a number of innovative approaches in conservation science. The most noteworthy is the adoption of multiple‐objective planning whereby, in addition to biodiversity, conservation plans account for everything from mining to wave energy.

**Figure 4:**
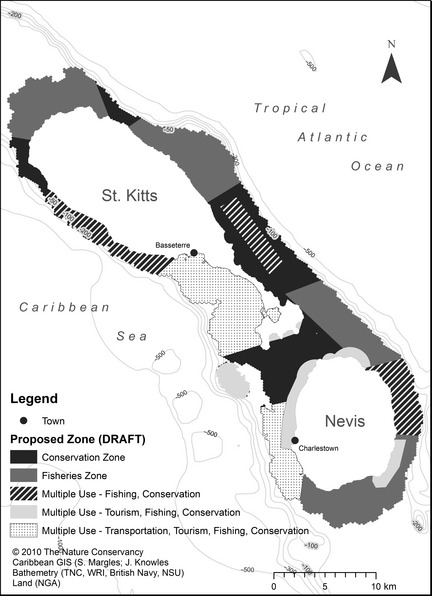
The results of a marine spatial planning exercise at St. Kitts‐Nevis, Caribbean. Using a decision support system, conservation planners are able to delineate zones for different uses such as fisheries, conservation, tourism and transportation. Modified from Agostini *et al*. (2010).

Multiobjective conservation approaches take the form of marine spatial planning that establishes zones of economic activity (Gleason *et al*. 2010), planning for alternative energy development (Cameron, Cohen & Morrison 2012), securing environmental flows through sustainable hydropower development (Richter & Thomas 2007), protecting groundwater‐dependent ecosystems (Brown *et al*. 2011) and conserving biodiversity on grazed ranchlands (Pyke & Marty 2005). These approaches implement conservation on relatively large expanses of lands and waters that are also being managed for human uses. In some cases, this requires partitioning out a landscape, river basin or seascape to achieve multiple objectives such as through marine zoning (Fig. 4). In other cases, the focus is on improving the management of an ecological process such as rates of stream flow. A related innovation is ‘development by design’, which uses a mitigation hierarchy to secure new conservation lands in exchange for development in areas of lesser conservation value. Development by design has led to the protection of millions of hectares of land in Mongolia and western United States in exchange for permitting oil and gas or mineral extraction in limited areas (Kiesecker *et al*. 2010). Multiobjective thinking leads naturally to the recognition that conservation must work in the context of values other than biodiversity, including especially concern for human well‐being (Kareiva 2014). As a result, TNC scientists have begun experimenting with strategies that pay increased attention to what nature does for humanity – or so‐called ecosystem services – in large part because these services to people provide a different, supplemental way to raise money and stimulate action for the protection of nature.

## Incorporating ecosystem services into conservation practice and planning

Millennium Ecosystem Assessment or MEA (2005) focused the world's attention on the many ways humanity relies on nature, not just for products, but for a huge range of services including clean water, flood regulation, regulation of climate‐warming carbon dioxide and protection of coastal communities from storm surge. The MEA also highlighted just how threatened many of these services had become over the prior 50 plus years. At the same time, economists were developing new tools to estimate the monetary value of nature. While attempts to put a price tag on nature's services have valid criticisms (e.g. Wunder 2013), the work did highlight approaches that might help policy‐makers as well as political and business leaders to appreciate the tangible costs to degrading nature.

The Nature Conservancy has become increasingly interested in strategies that better align people's individual incentives with what is good for both nature and the broader human community. One such strategy involves Payments for Ecosystem Services projects, whereby governments or other parties financially reward landowners for conserving and restoring the flow of ecosystem services (Jack, Kousky & Sims 2008). In this vein, TNC, in partnership with the U.S. Agency for International Development and local Ecuadorian groups, established in 2000 a water fund that is paid into by the Quito Municipal Water and Sewage Agency, the Quito Electricity Company and the Andina Beer Company. The fund has been used to plant millions of trees within the city's watershed, support hydrologic modelling and monitoring and hire new guards to improve enforcement of restrictions on logging and grazing within the Condor Bioreserve upstream of the city (Krchnak 2007). TNC seeks to replicate this level of success by establishing water funds around the world (Goldman‐Benner *et al*. 2012). In all cases, some funds are directed to promoting alternative, less environmentally damaging livelihoods for communities in the targeted watersheds.

Along with Stanford University, World Wildlife Fund and the University of Minnesota, TNC co‐initiated in 2006 a collaboration known as the Natural Capital Project, or NatCap (http://www.naturalcapitalproject.org/). NatCap aims to create straightforward, user‐friendly models that link changes in the delivery of ecosystem services directly to changes in the use and management of lands and waters (Kareiva *et al*. 2011). By providing these models and training to local experts, NatCap scientists help leaders to more fully appreciate and explicitly weigh into their decisions the many benefits from natural ecosystems. The NatCap scientists have worked with local experts in 20 nations on projects ranging from spatial planning to programmes that establish Payments for Ecosystem Services (Ruckelshaus *et al*. 2013). One lesson emerging from NatCap's suite of projects is decision‐makers rarely request that all of nature's benefits be reduced to a single common currency or ‘dollar value’ in order to weigh their value to society (Ruckelshaus *et al*. 2013).

**Figure 5:**
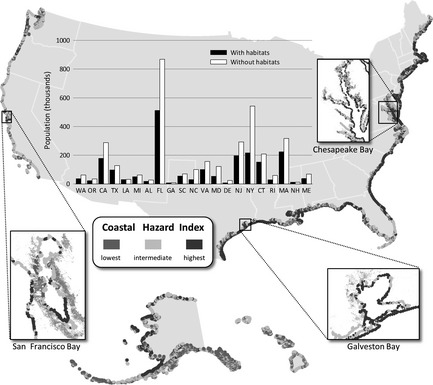
Protection from storm surge provided by coastal habitats. Warmer colours indicate regions anticipated in 2100 to have the greatest exposure to storm hazards, given anticipated sea level rise and other impacts of climate change. In the inset graph, black bars show the number of people in each coastal state living in areas most exposed to hazards (red areas in the map) with protection provided by habitats. White bars show the larger numbers of people that would be exposed to this same high level of risk if habitats were lost due to climate change or human impacts. Reprinted from Arkema *et al*. (2013), first published in *Nature Climate Change*.

The Nature Conservancy scientists are pioneering efforts to incorporate potential benefits to people into the conservation planning process. For instance, they are accounting for ecosystem services in their plans to conserve coastal ecosystems. These ecosystems such as oyster reefs, saltmarshes, coral reefs, sea grass beds and mangrove forests provide considerable protection of coastal communities. Beck *et al*. (2011) have prioritized areas restoring oyster reefs and their ecosystem services, and their analyses are helping guide on‐the‐ground reef restoration activities (http://www.nature.org/ourinitiatives/habitats/oceanscoasts/howwework/restoration-works-oyster-reefs.xml). Mark Spalding and colleagues (2013) synthesized the substantial data that can be used to predict when these coastal ecosystems will play an important role in attenuating storm surge and preventing coastal erosion. Complementing this effort, Arkema *et al*. (2013) mapped the degree to which coastal habitats reduce the risk to human life and property in North America (Fig. 5). Accounting for anticipated sea level rise, they found that the number of people, poor families and elderly individuals projected to be most at risk of hazards can be reduced by half if existing coastal habitats remain fully intact. Similar analyses, but performed on a smaller spatial scale, have been used to determine the design and location of a large oyster reef restoration project in Mobile Bay, Alabama, with the goal of maximizing coastal protection benefits (http://www.nature.org/ourinitiatives/regions/northamerica/unitedstates/alabama/explore/main-page-mobile-bay-restoration.xml). The larger‐scale analyses are too recent to have yet impacted actions on the ground, but they will guide TNC's future investment in coastal restoration and how those projects take shape.

## Engaging corporations as conservation actors

Like most other conservation NGOs, TNC has increasingly turned to working with the corporate and finance sectors to advance conservation goals (Robinson 2012). TNC's work with corporations stems from analyses demonstrating that global corporations contribute significantly to land conversion and land and water degradation that threaten biodiversity. For example, Coca‐Cola uses the equivalent of 3000 Olympic‐sized swimming pools of water every day just for production, and Rio Tinto has mining permits globally for lands with a cumulative footprint the size of the U.S. state of Montana. Given their role in the earth's ecology, global corporations provide an opportunity to reduce ecosystem degradation if corporate practices can be altered in a way that minimizes biodiversity loss.

While it might seem naïve to expect corporations to become allies in conservation efforts, recent trends indicate otherwise. A remarkable percentage of the average corporation's value can be attributed to intangible assets, such as brand and corporate reputation. The Ocean Tomo Intangible Asset Market Value study indicates that this intangible value has increased to 80% in 2010, from a mere 17% in 1975 (Ocean Tomo 2010). The relevance to conservation is that companies are increasingly paying serious attention to sustainability, and reputation provides a lever with which to influence corporations to take conservation seriously. For this reason, TNC is working with over 50 major companies, including Rio Tinto, Coca‐Cola, Cargill, General Mills, WalMart and Shell (http://www.nature.org/about-us/working-with-companies/companies-we-work-with/index.htm). TNC has also worked closely with industry associations such as the Alliance for Water Stewardship (http://www.allianceforwaterstewardship.org) and the Forest Stewardship Council (FSC) (http://us.fsc.org/) and, in the case of FSC, has assisted in the development of their standards.

The Nature Conservancy's collaborations with corporations sometimes entail developing scientific tools and analyses to help companies incorporate the value of nature into their business decisions. The most developed example of this is a partnership with Dow Chemical Company, whereby TNC scientists have analysed the role of natural habitats in reducing ground‐level ozone, preventing storm damage and regulating water flows and quality. As a result, Dow is considering investing in hardwood forest restoration as a way of reducing ozone in the Houston area (e.g. TNC & Dow 2012). This research is at an early stage, but the hope is that Dow will eventually routinely factor the value of coastal habitats, watersheds and forests into its business decisions and sustainability goals (Molnar & Kubiszewski 2012).

## ‘Conservation as negotiation’ places new demands on TNC science

Science is central to TNC's culture. Virtually, all of its major field programmes, global projects and strategies have scientists as members. Nearly every major initiative in TNC has a transparent science underpinning. There is an increasingly strong sense that publishing a peer‐reviewed study is an important way to validate new ideas and share those ideas within the conservation community. But there remains room for improvement.

Most notably, after investing millions of dollars and almost 10 years into ecoregional plans and assessments, it became clear that simply identifying high‐priority places and actions was not enough. As a result, TNC has embraced the need to expand the definition of a conservation scientist to include social scientists, economists and communication experts. Because the politics of conservation inevitably entails some compromise, there is also pressing need for better science to inform those compromises. Modern conservation is as much about managing resource use and extraction as it is about setting aside protected areas. The biggest challenge is knowing when another mine, or another oil pad, or another hundred hectares of heavily fertilized crops is too much and thus will jeopardize both biodiversity and ecosystem services. Ecological theory reveals that thresholds and tipping points are inherent in complex nonlinear systems (Scheffer *et al*. 2012). But the science is lacking for anticipating where those thresholds are and how to account for cumulative impacts. The ecology of cumulative risks, resilience and thresholds, in addition to tried and true land and water protection methods, holds the key to conservation success in the Anthropocene.

A second major challenge for conservation entails getting the greatest impact for one's investment. Foundations and donors increasingly want to know that their support is yielding results (Tierney & Fleishman 2011). ‘Good intentions’ or even ‘doing good’ is not enough. Conservation scientists have developed practical methods for estimating returns on investment for different conservation interventions (Murdoch *et al*. 2007), and these methods need to be more routinely deployed in conservation practice. Then, following a conservation intervention, there needs to be systematic assessment of outcomes – especially when promises are made about delivery of clean water or improved human well‐being. Despite significant investment in monitoring of interventions over the last decade, TNC and most conservation organizations still have further to go in understanding the degree to which most conservation actions are really working (Muir 2010).

**Figure 6:**
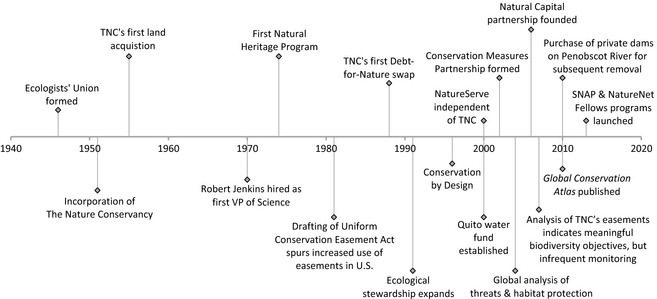
A timeline highlighting the intertwining of major innovations in both conservation science and conservation practice at TNC.

Much of conservation depends on politics, leadership, marketing and the opportunity provided by some tranche of public or private funding. These enabling conditions can be as or more important than detailed scientific analyses. There are also some practical limitations of science in conservation. For example, climate change, ocean acidification, exotic species, extreme weather, political and social upheavals and the many other forms of ecological and socioeconomic volatility challenge our skills at prediction. Given these constraints and the importance of enabling conditions for conservation, the most effective conservation results will be achieved through a mix of opportunism, experience such as that provided through the Conservation Coaches Network and science. One certainty is that use‐inspired conservation science must continually evolve and sometimes embrace entirely new disciplines to keep pace with environmental change and threats to biodiversity (Fig. 6).

## The future: building partnerships to support use‐inspired conservation science

Graduate students around the world seek to frame their ecological research in today's urgent questions about extinction, loss of ecosystem services and global climate change. In short, there is a huge appetite for use‐driven science and stronger linkages between resource management agencies, NGO science and university science. Still lacking, however, are the institutions to transform this enthusiasm into action. In response, TNC has launched two new science initiatives to address this institutional gap to some degree. The first is SNAP (Science for Nature and People – see http://www.snap.is), a partnership of the National Center for Ecological Analysis and Synthesis, WCS and TNC aimed at providing rapid, implementable solutions for conservation challenges that threaten the integrity of nature and human well‐being. TNC and WCS helped create SNAP because on their own they cannot produce the science needed to address these challenges. SNAP affords these organizations a way to engage a global network of scientists and experts in academia, the private sector and government agencies to address some of the world's biggest issues.

As a complement to SNAP, NatureNetFellows (http://www.nature.org/ourscience/naturenet-science-fellowship.xml) is an international postdoctoral programme that seeks to cultivate skill sets such as engineering and scenario modelling that are needed to advance conservation in the Anthropocene. This new fellowship programme will complement an existing postdoctoral programme – the David H. Smith Conservation Research Fellowship Programme (http://www.conbio.org/mini-sites/smith-fellows) that is managed by the Society for Conservation Biology but which TNC helped launch. TNC is not the only conservation organization to recognize the need for better links to academic centres of excellence. Recently, the Luc Hoffman Institute was launched ‘to connect research capacity and multidisciplinary thought leaders from around the world with WWF's global network of practitioners and scientists' (http://luchoffmanninstitute.org/). Meanwhile, several academic conservation programmes offer internship programmes that seek to give their students real‐world experience in conservation NGOs. These initiatives will help TNC and other organizations to bridge the gap between theory and practice and take better advantage of existing scientific expertise to solve conservation problems.

Science can best serve on‐the‐ground conservation when it helps to influence priorities and actions and is, in turn, improved through application of science to conservation action. At the same time, we recognize that scientific analyses and information are not a panacea for conservation. Ultimately, conservation success will depend on changing values and behaviour. Social science and cognitive psychology can help conservationists understand what makes people change their behaviour or beliefs. In the end, leadership and communication will be every bit as essential as science.
